# Biographic Clinics

**Published:** 1904-06

**Authors:** 


**Biographic Clinics.** By George M. Gould, M.D. Vol. II. Pp. xiii., 9–392. London: Rebman Ltd. 1904.

It is only a few months since we noticed the first volume of these clinics (vol. xxi., p. 358). The industry of Dr. Gould has enabled him to follow on quickly with an account of the origin of the ill-health of George Eliot, George Henry Lewes, Wagner, Parkman, Jane Welch Carlyle, Spencer, Whittier, Margaret Fuller Ossoli, and Nietzsche. We are informed that the journalists and reviewers have not yet placed the facts adequately before the profession, and "the truth is too important to the profession and to the world, especially to its literary workers, to allow the neglect of the past generation to be continued for another. I am entirely convinced that instead of exaggeration I have under-estimated the tremendous importance and extent of the morbid factor called eyestrain. The profession must be made to realise the enormity of the mistake it has made in ignoring the truth." Much is to be said in favour of Dr. Gould's views, and this volume contributes a large amount of interesting personal history, tending to give some support to the view that eyestrain is responsible for much of the ubiquitous migraine, brain-fag, melancholy, morbidity, and despair which have been so apt to affect the world's great writers. "All is chaos, ignorance, and incurability, . . . and yet a few drops of a mydriatic in the eyes will cure it, temporarily of course, and non-use of the eyes will prevent it; a wrong pair of spectacles will cause it, and right ones will cure it." What a field opens up to the neurologists and ophthalmologists, and yet they are not grateful. The first chapter on eyestrain and the literary life is a study of the disease of fourteen literary patients who lived in the last century. "In varying admixture and degrees the symptoms of these patients were headache, insomnia, 'biliousness,' sick-headache, 'nervousness,' dejection, indescribable suffering, inability to do literary work without producing these symptoms, and relief of the symptoms whenever, even for a day or a few hours, literary work was stopped, and entire cessation of the characteristic symptoms at about 60 years of age." . . . "Much, if not the most of it, is due to neglect of the physiology of the eye and of its reflex neuroses, and carelessness as to the functional diseases which depend upon eyestrain. The deadliest blow that can be given to quackery in and out of the profession, to the patent medicine and eddyistic humbugs, is to prevent the dyspepsias, anæmias, neurasthenias, and headaches which are caused by eyestrain, and whereon fatten the multitude of quacks." We have allowed the author to speak for himself, as he is well able to do. The book is one to be studied, and it concludes with an appendix giving sixty-eight reasons why glasses have not more frequently given relief.

